# Metabolomics Insights of the Immunomodulatory Activities of Phlorizin and Phloretin on Human THP-1 Macrophages

**DOI:** 10.3390/molecules26040787

**Published:** 2021-02-03

**Authors:** Noelia Cambeiro-Pérez, Xiana González-Gómez, Carmen González-Barreiro, María Rosa Pérez-Gregorio, Iva Fernandes, Nuno Mateus, Victor de Freitas, Borja Sánchez, Elena Martínez-Carballo

**Affiliations:** 1 Department of Analytical and Food Chemistry, Facultade de Ciencias, Universidade de Vigo, Campus da Auga, 32004 Ourense, Spain; ncambeiro@uvigo.es (N.C.-P.); xgonzalez@uvigo.es (X.G.-G.); cargb@uvigo.es (C.G.-B.); 2 Department of Chemistry and Biochemistry, Faculty of Sciences, LAQV/REQUIMTE, University of Porto, E-4169-007 Porto, Portugal; iva.fernandes@fc.up.pt (I.F.); nbmateus@fc.up.pt (N.M.); vfreitas@fc.up.pt (V.d.F.); 3 Department of Microbiology and Biochemistry, Dairy Research Institute of Asturias, Spanish National Research Council (IPLA-CSIC), Paseo Río Linares sn, 33300 Villaviciosa, Spain; borja.sanchez@csic.es

**Keywords:** untargeted metabolomics, THP-1 macrophages, phloretin, phlorizin, immunomodulation

## Abstract

Dihydrochalcones, phlorizin (PZ) and its aglycone phloretin (PT), have evidenced immunomodulatory effects through several mechanisms. However, the differential metabolic signatures that lead to these properties are largely unknown. Since macrophages play an important role in the immune response, our study aimed to characterise human THP-1 macrophages under PZ and PT exposure. A multiplatform-based untargeted metabolomics approach was used to reveal metabolites associated with the anti-inflammatory mechanisms triggered by the dihydrochalcones in LPS-stimulated macrophages, for the first time. Results showed differential phenotypic response in macrophages for all treatments. Dihydrochalcone treatment in LPS-stimulated macrophages mimics the response under normal conditions, suggesting inhibition of LPS response. Antagonistic effects of dihydrochalcones against LPS was mainly observed in glycerophospholipid and sphingolipid metabolism besides promoting amino acid biosynthesis. Moreover, PT showed greater metabolic activity than PZ. Overall, the findings of this study yielded knowledge about the mechanisms of action PZ and PT at metabolic level in modulating inflammatory response in human cells.

## 1. Introduction

Phlorizin (PZ) and its aglycone phloretin (PT), the major dihydrochalcone flavonoids found in apples, have been shown to exert beneficial effects on the human health such as antioxidant potential [1], anti-cancer effects [2,3] and immunomodulatory activities both in vitro and in vivo [4]. Since macrophages are the most critical cells in initiation, maintenance, and resolution of inflammation [5], dihydrochalcone effects on these cells have been evaluated. They are a heterogeneous population of cells with high ability to acquire distinct functional phenotypes in response to different environmental changes and stimuli [6]. Activation signals include cytokines such as interferon gamma (IFN-γ), granulocyte-monocyte colony stimulating factor (GM-CSF), and tumor necrosis factor alpha (TNF-α); bacterial lipopolysaccharide (LPS); extracellular matrix proteins; and other chemical mediators. Activated macrophages are deactivated by anti-inflammatory cytokines such as interleukin 10 (IL-10) and transforming growth factor beta (TGF-β); and cytokine antagonists that are mainly produced by macrophages. Therefore, macrophages participate in an autoregulatory process necessary for maintaining the tissue homeostasis [5].

Chang et al. [7] evidenced the anti-inflammatory effects of PT by reducing levels of proinflamatory cytokines and mediators (NO, PGE2, IL-6, TNF-α, iNOS and COX-2) in LPS-stimulated murine RAW264.7 macrophages whilst PZ do not exert inhibitory effects. Chauhan et al. [8] also suggested that PT targeted the TLR4-NF-κb pathway to suppress *Escherichia coli* K1-induced inflammation in RAW264.7 macrophages. Zhao et al. [9] evaluated immunomodulatory activities of PZ metabolites also in LPS-stimulated RAW264.7 cells, demonstrating that PZ structural form is crucial for the development of anti-inflammatory activity since the metabolites presented differential effects on NO production, iNOS mRNA expression, iNOS protein expression, TNF-α, IL-10, VEGF, CCL2 and CXCL1 mRNA expression. Another study yielded knowledge about the anti-inflammatory activity of docosahexaenoic acid ester of phloridzin (PZ-DHA) in LPS-stimulated human THP-1 macrophages by reducing TNF-α, IL-6 and COX-2 [10]. Antiobesity effects of PZ and PT by modulating the relationship between 3T3-L1 adipocytes and RAW 264.7 macrophages were assessed, revealing that PT is more effective than PZ towards this term [4,11]. Thus, all these insights reinforce the importance of the phenolic compound structure in modulating immune activity.

According to literature, most of the in vitro studies on the anti-inflammatory capacity of these two dihydrochalcones have been performed in the murine macrophage cell line RAW 264.7, evaluating its ability to modulate the production of cytokines and mediators of the inflammatory process. Nevertheless, there are no previous works that analyse their role in modulating the intracellular metabolism of human macrophages. Given the heterogeneity of macrophages, and taking into account that metabolism is closest to the phenotype, it is important to generate knowledge about how PZ and its aglycone PT modulates the metabolic status of human macrophages.

Although Abuawad et al. [6] studied the characterisation of THP-1 macrophage polarization through a metabolomics approach, to the best of our knowledge, there are no previous studies that report functional biological readouts about the effects of PZ and/or PT. Preliminary works of our research group demonstrated how untargeted metabolomics constitutes a powerful tool for biological interpretation of molecular and metabolic changes induced by proteinaceous molecules in human immune cells [12,13].

In this study, untargeted multiplatform metabolite profiling by LC-MS and GC-MS was conducted not only to characterise human THP-1 macrophages phenotypes under PZ and PT exposure but also to reveal metabolic signatures associated with the anti-inflammatory mechanisms triggered by the dihydrochalcones in LPS-stimulated THP-1 macrophages. Multiplatform metabolomics approaches cover a wider spectrum of compounds than single-platform approaches, providing a fuller picture of the cell status. GC-MS can be used to analyse volatile and semivolatile metabolites with small molecular weight (e.g., amino acids, nucleic acids, bile acids, and saccharides) while LC-MS covers large hydrophobic metabolites, mainly lipids.

The generation of knowledge about the mechanisms of action of naturally occurring compounds for the modulation of the inflammatory response in human cells, is growing in significance to find potential therapeutic targets for the treatment of inflammatory diseases.

## 2. Results and Discussion

To evaluate the immunomodulatory role of dihydrochalcones through metabolomics, TPA-differentiated human TPH-1 macrophages were cultured with dihydrochalcones standards (phlorizin; PZ and phloretin; PT). To assess the possible antagonistic effects of both dihydrochalcones to lipopolysaccharide (LPS), a well-known pro-inflammatory agent, metabolomics profiling of THP-1 derived macrophages following LPS, PZ + LPS (PZL) and PT + LPS (PTL) treatments was also performed. Cell viability was not significantly altered by the doses of treatment compounds used in this study by MTT assay (data not shown).

### 2.1. Overall Effect of Experimental Conditions on Macrophage-Like THP-1 Cells Metabolome

**Figure 1:**
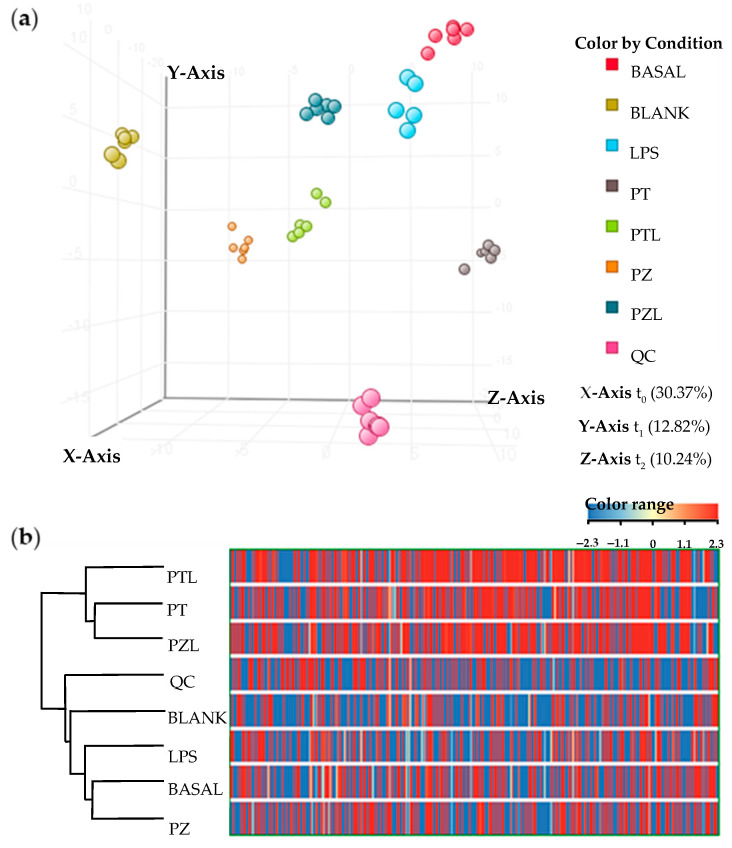
Multivariate statistical analysis of the subsequent evaluated conditions: Basal; LPS, lipopolysaccharide; PZ, phlorizin; PT, phloretin; PZL, phlorizin + LPS; PTL, phloretin + LPS; Blank and QC, quality control. (**a**) PLS-DA score plot (accuracy of the model of 91.254%, R_2_ = 0.567 and Q_2_ = 0.167); (**b**) Hierarchical clustering analysis by applying Pearson’s centered absolute similarity measure and complete linkage.

After combined LC and GC data processing by MPP, 507 entities were significantly differentiated (*p*-value < 0.050 and fold change > 2.0) among conditions (BASAL, BLANK, LPS, PT, PTL, PZ, PZL and QC). Data showed a grouped distribution without outlier samples in the partial least squares-discriminant analysis (PLS-DA), the highest fraction of *explained variance* among the three first principal components (accuracy of the model of 91.254%, R_2_ = 0.567 and Q_2_ = 0.167) was 53% (Figure 1a). According to analytical method validation parameters for quality control, the lower dispersion among QC samples observed in the PLS-DA, provides a reliable stability and repeatability throughout the entire sequence. Moreover, blank samples were also clustered and separated from the other groups. Hierarchical clustering analysis was performed by applying Pearson’s centered absolute similarity measure and complete linkage, to emphasize the relationship among conditions, which revealed two main clusters: PZL-PT-PTL and LPS-BASAL-PZ (Figure 1b).

**Figure 2:**
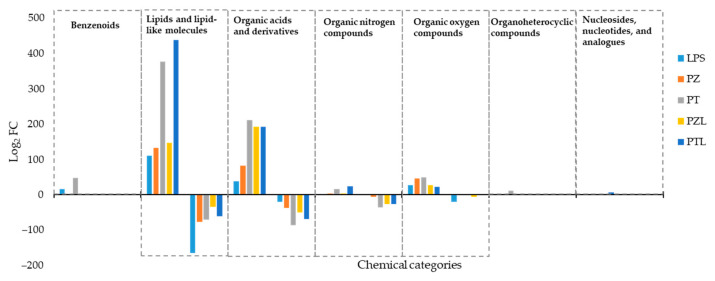
Specific chemical categories of dysregulated entities in the evaluated conditions (LPS, lipopolysaccharide; PZ, phlorizin; PT, phloretin, PZL, phlorizin + LPS; PTL, phloretin + LPS) compared to untreated THP-1 differentiated macrophages (BASAL). Increased and decreased entities were indicated by the fold change (FC > 2.0) and were also adjusted to *p*-values < 0.05.

To deepen the knowledge of these clustering, moderated *t*-tests with FDR correction were applied to identify the entities responsible for significant differences among treated and untreated cells. Taking into account its chemical classification, it was observed that those entities differentially produced by treated THP-1 with respect to the untreated cells were, to a greater extent, lipids and lipid-like molecules (mainly fatty acids and conjugates, fatty amides, glycerophosphoglycerols, monoradylglycerols and phosphate esters), followed by organic acids and derivatives (mainly amino acids, peptides, and analogues) as it was represented in the bar charts of Figure 2. However, considering organic acids and derivatives, changes were less pronounced in LPS treated cells compared to the other conditions. Similarly, according to the lipids and lipid-like molecules category, LPS treated cells showed a differential behaviour with respect to the other conditions with a predominant down-regulated response while the rest showed an up-regulated predominant pattern.

Both lipid and amino acid metabolism were reported to play an important role in immune response. Lipids, contributes to regulate macrophage and T lymphocyte function and phenotype by fulfilling its energetic needs and regulating membrane fluidity [14]. Whilst amino acids were involved in the activation of lymphocytes, macrophages and natural killer cells; cellular redox state; gene expression; lymphocyte proliferation; and the production of antibodies, cytokines and other cytotoxic substances [15].

### 2.2. Differential Metabolomic Profile of Dihydrochalcones and LPS on Macrophage-Like THP-1 Cells

**Figure 3:**
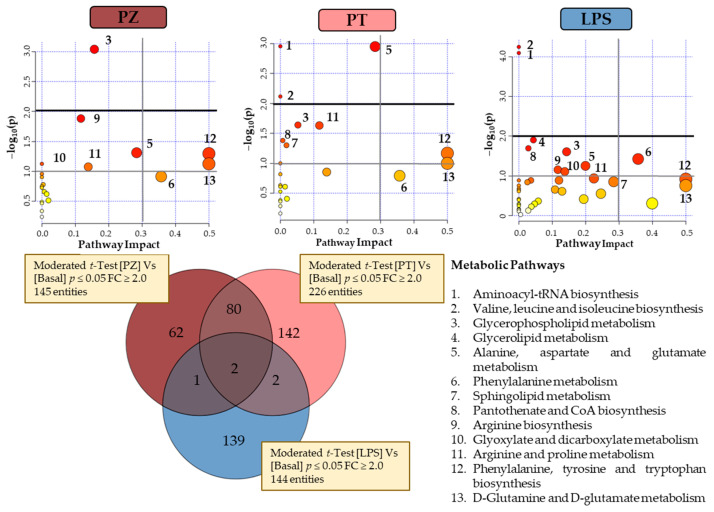
Venn diagram and metabolome overviews obtained from MetaboAnalyst pathway analysis of the subsequent evaluated conditions: PZ, phlorizin; PT, phloretin; and LPS, Lipopolysaccharide compared to BASAL. The top-pathways are ranked by the gamma-adjusted *p* values for permutation per pathway (y-axis) and the total number of hits per pathway (x-axis). The colour graduated from white to yellow, orange and red, circle size (large > small) as well as the values of both x and y increase represents the degree of significance.

Figure 3 shows a Venn diagram with the significantly changed entities in THP-1 macrophages treated with PZ and PT, as well as with LPS, compared to untreated cells (BASAL), to evaluate the effect to the pro-inflammatory phenotype of LPS-treated macrophages. Univariate *t*-test with FDR correction revealed 145 and 226 total entities to PZ and PT, respectively. In particular, 62 and 142 entities specific to PZ and PT, respectively. Regarding to LPS treatment, Venn diagram revealed 139 specific entities. To understand the biological implications of these alterations, an analysis of metabolic pathways was performed by MetaboAnalyst 4.0 [16] through pathways analysis tool. In the summary of pathway analysis illustrated in Figure 3 it was observed that PZ mainly modifies glycerophospholipid metabolism and aminoacyl-tRNA biosynthesis while PT modifies to a greater extent alanine, aspartate and glutamate metabolism.

According to the observed in both clustering and PCA explained above, the behaviour of PT-treated cells seems to be the most differential with respect to other two conditions. However, considering the few common entities between LPS and each dihydrochalcone (1 with PZ and 2 with PT), there is statistically significant evidence that suggests a differential response for each condition. LPS cells showed a predominant alteration in valine, leucine and isoleucine biosynthesis and aminoacyl-tRNAbiosynthesis pathways (Figure 3).

#### 2.2.1. Amino Acid Metabolism

Alanine, aspartate and glutamate metabolism were all significantly altered in treated THP-1 derived macrophages. More specifically, cells treated with PT show the greatest alteration of this pathway with the significantly increase of *N*-methylglutamic acid, L-glutamic acid, *N*-acetyl-L-aspartic acid and L-asparagine besides the remarkable decrease of D-aspartic acid (Table S1). Alterations in this pathway were reported by Abuawad et al. [6] in anti-inflammatory phenotype of THP-1 derived macrophages. This is consistent with the metabolome plot from pathway analysis (Figure 3), where this pathway is less relevant in LPS-treated cells than in the other two conditions. Decrease in D-aspartic acid level could be explained by its consumption in a reaction with acetyl-CoA to produce *N*-acetyl-L-aspartic acid by L-aspartate *N*-acetyltransferase. However, LPS and PZ treated cells showed no alteration in the production of D-aspartic acid but did of *N*-acetyl-L-aspartic acid, that could be explained as the fold change for *N*-acetyl-L-aspartic was lower in LPS and PZ-treated cells compared to PT exposed cells. Since *N*-acetyl-L-aspartic acid can be converted to glutamate in neuronal tissue, it could act as a reservoir for replenishing glutamate during times of stress and/or energy demand [17]. Glutamate could feed into TCA cycle to be converted to α-ketoglutarate by glutamate dehydrogenase. However, no significant alterations have been observed in the intermediaries of this metabolic pathway nor related, for any of the studied conditions although there was reported in the literature to be hallmarks for M1 pro-inflammatory macrophages (glycolysis and pentose phosphate pathway) in LPS-treated cells and for M2 anti-inflammatory macrophages (OXPHOS which connected to TCA cycle). Nevertheless, our results were consistent with those reported in THP-1 polarized macrophages by Abuawad et al. [6]. They suggest that these observations could be due to macrophages heterogeneity, the polarization stimuli and the selected in vitro culture (primary or cell line from different species, tissues or progenitors) what may entail different biological and metabolic signatures.

Valine, leucine and isoleucine biosynthesis; arginine biosynthesis and aminoacyl-tRNA biosynthesis were also found modulated by LPS, PZ and/or PT treatment, being in this last condition in which the changes related to the metabolism of amino acids predominate. Specifically, valine, leucine and isoleucine biosynthesis was found mainly altered by PT treatment with the up-regulation of L-valine and L-leucine. In the case of arginine biosynthesis, all conditions showed up-regulation both urea and L-glutamate, although PT treated cells showed highest FC levels. In the same way, alterations in aminoacyl-tRNA biosynthesis were more relevant in PT treated cells due to the up regulation of eight aminoacids (glycine, L-asparagine, L-isoleucine, L-leucine, L-glutamate, L-phenylalanine, L-threonine and L-valine), compared to PZ (glycine, L-isoleucine, L-glutamate, L-phenylalanine and L-threonine). Generally, these results suggest that PT promotes an increased protein synthesis rate through up-regulation of amino acid biosynthesis. Moreover, it is known that amino acids affect immune responses either directly or indirectly through their metabolites [15].

#### 2.2.2. Lipid Metabolism

Glycerophospholipids (GLs) are components of cell membrane and lipoproteins which are involved in multiple biological processes, such as inflammation and cell differentiation [18]. As Table S1 shows among the metabolites involved in the glycerophospholipid metabolism, entities dysregulated by LPS, PZ and/or PT treatment compared with the untreated cells: phosphatidylcholines (PC), phosphatidylglycerols (PG), phosphatidylethanolamines (PE), and sn-glycero-3-phosphocholine were found. PC were significantly downregulated in both dihydrochalcone treated cells while LPS showed no statistically significant changes. PG were significantly upregulated only in PT-treated cells, while no statistically significant differences were observed in PZ-treated cells. In the case of LPS-treated cells, PG(15:0/16:0) was found downregulated and PG(15:0/18:2(9Z,12Z)) upregulated. PE were found upregulated by LPS whilst by dihydrochalcone were found both up and downregulated. Enhancement of sn-glycero-3-phosphocholine was exclusively observed in the case of cells treated with LPS. Zhang et al. [18] studied the lipid profiles of human M1 (pro-inflammatory phenotype) and M2 (anti-inflammatory phenotype) macrophages derived from THP-1. They observed a general trend in glycerophospholipids metabolism, in which PC and PE levels where found increased in M1 whilst in M2 these levels where found decreased and PG levels increased. Moreover, there are scientific evidences relating the choline phospholipid metabolism and macrophage immune responsiveness [19]. In fact, a recent study have found that reducing *de novo* PC biosynthesis rate in macrophages alleviates white adipose tissue inflammation and insulin resistance in obese mice [20].

Polarization of human macrophages was associated with differential regulation of sphingolipid mediators, sphingosine, and ceramide kinases [21]. In the present study, alterations in sphingolipid metabolism were observed mainly in LPS-stimulated THP-1 derived macrophages with both up and downregulation patterns of ceramides and the upregulation of *O*-phosphocolamine. PT-treated cells showed few alterations in ceramides besides upregulation of the ganglioside NeuGcalpha2-3Galbeta1-4GlcNAcbeta1-3Galbeta1-4GlcNAcbeta1-3Galbeta1-4Glcbeta-Cer(d18:1/16:0) and *O*-phosphocolamine, which was found downregulated in PZ-treated cells (Table S1).

In particular, lipid metabolism contributes to macrophage phagocytosis by fulfilling its energetic needs and regulating membrane fluidity necessary for this process [14]. There are evidences about the bidirectional interaction between sphingolipids and glycerophospholipids, in opposed directions, contributing to membrane lipid homeostasis and lipid signalling events. Specifically, it was reported that ceramides reduce the levels of PE and prevent the biosynthesis of PC [22], which is consistent with the results observed in the present study.

### 2.3. Effects of Dihydrochalcones on LPS-Stimulated Macrophage-Like THP-1 Cells

**Figure 4:**
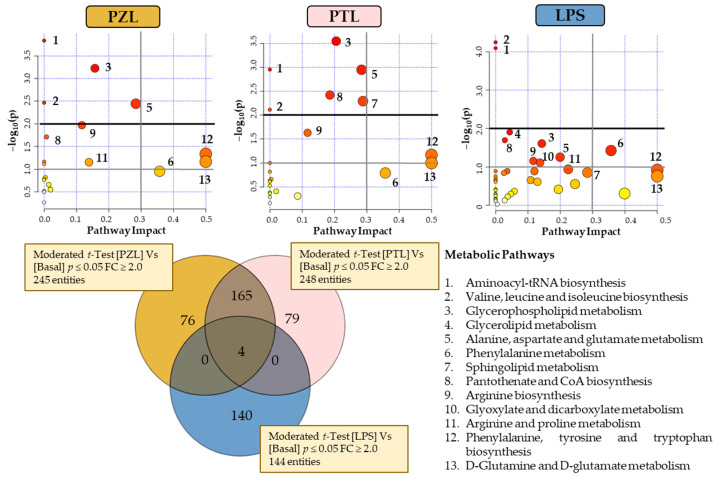
Venn diagram and metabolome overviews obtained from MetaboAnalyst pathway analysis of the subsequent evaluated conditions: PZL, phlorizin + LPS; PTL, phloretin + LPS; and LPS compared to BASAL. The top-pathways are ranked by the gamma-adjusted *p* values for permutation per pathway (y-axis) and the total number of hits per pathway (x-axis). The colour graduated from white to yellow, orange and red, circle size (large > small) as well as the values of both x and y increase represents the degree of significance.

Metabolomics profiling of the TPA-differentiated THP-1 cells following LPS, PZ + LPS (PZL) and PT + LPS (PTL) treatments were also examined to assess the possible antagonistic effects of phloretin and phlorizin to LPS. A Venn diagram with the significantly changed entities is presented in Figure 4. As can be seen, different behaviour has been observed with 245 and 248 total entities to PZL and PTL have been revealed, respectively. Using the analysis of metabolic pathways when LPS-stimulated macrophages treated with PZ and untreated macrophages were compared, modulation on aminoacyl-tRNA biosynthesis; glycerophospholipid metabolism; valine, leucine and isoleucine biosynthesis and alanine, aspartate and glutamate metabolism were observed. In the same way, the comparison of LPS-stimulated macrophages treated with PT and untreated macrophages (Figure 4), also revealed the modulation of glycerophospholipid metabolism; aminoacyl-tRNA biosynthesis; alanine, aspartate and glutamate metabolism and valine, leucine and isoleucine biosynthesis; besides alterations in pantothenate and CoA biosynthesis and sphingolipid metabolism.

It was observed that cells treated with LPS + dihydrochalcone showed similar response pattern to those cells just stimulated with only phenolic compounds. Therefore, these results suggest that dihydrochalcones could be capable of revert the response triggered by LPS. Nevertheless, comparing the metabolic activity of PZ and PT it is noticeable that their modulation has been quite different. In fact, an antagonistic effect of PT against the pro-inflammatory metabolism promoted by LPS was clearly observed in glycerophospholipid metabolism, as well as it was also highly remarkable in sphingolipid metabolism. However, minimal metabolic differential activity from untreated condition was reported by PZ, despite also being antagonistic against LPS. Results also suggest an increased amino acid biosynthetic rate in both LPS + dihydrochalcone treated cells compared to LPS.

There are evidences that both PT and PZ have been shown to exert immunomodulatory and anti-inflammatory activities as discussed earlier [4]. Nevertheless, it is noteworthy that the type and position of sugar moiety can impact the immunosuppressive activity of phenolic compounds [23]. Several studies have also reported a differential effect between PZ and its aglycone derivative. Ehrenkranz et al. [24] reviewed the different pharmacological activities between PZ and PT. Huang et al. [11] results showed a superior anti-inflammatory activity of PT in macrophages stimulated by differentiated media from 3T3-L1 cells compared to PZ. In the same way, PT was found to significantly inhibit the levels of pro-inflammatory cytokines and mediators in LPS-stimulated murine RAW264.7 macrophages, while no suppression was observed by the glycosylated form [7]. Liddle et al. [4] also reported the stronger effectiveness of PT compared to PZ towards anti-inflammatory and angiogenic capacities by using in vitro models designed to reproduce the obese adipose tissue microenvironment. Therefore, aglycone moiety of PZ seems to be essential for its biological activities, so that PZ’s chemical structure modifications were reported to be necessary for its inhibitory role in inflammation [25].

The aglycones polyphenols, due to its lower molecular weight and higher hydrophobicity, are able to diffuse through the lipid bilayer whilst glycosylated forms, requires membrane-associated carrier protein active transport. For this reason, the first step of the intestinal metabolization process of the glycosylated polyphenols seems to be the cleavage of glucose moiety. Specifically, the hydrolysis of PZ to PT is performed by the lactase-phlorizin hydrolase, present on the brush border of the enterocytes [23]. However, lactose-intolerant individuals would be expected to have decreased PZ to PT conversion and no other pathways for the metabolism of PZ have been determined [24]. Although, glucose transporters have been reported to be involved in sensing and uptaking intact flavonoid glycosides, are still lacking comprehensive studies about the mechanisms of glycoside forms transport by cellular systems. Therefore, the bioavailability of phenolic compounds is clearly compromised by the glycosylation pattern, which has an impact on their metabolic effects and health benefits [26]. Based on all this, the differential metabolic effects observed between PZ and PT could be explained due to the time imbalance that the different cellular transport mechanisms involved entail besides the enzymatic cleavage of the sugar moiety.

## 3. Materials and Methods

### 3.1. Chemicals, Solutions and Materials

The human monocyte-like cell line THP-1 (#88081201) was obtained from the European Collection of Authenticated Cell Cultures (ECACC, Wiltshire, UK). RPMI 1640 medium containing L-alanylglutamine and sodium bicarbonate (Sigma-Aldrich, Madrid, Spain) was supplemented with 10% foetal bovine serum (FBS) (ICN Flow; Costa Mesa, CA, USA) and 1.0% of an antibiotic/antimicotic solution containing 100 U mL^−1^ of penicillin, 100 µg mL^−1^ of streptomycin and 0.25 µg mL^−1^ of amphotericin B from Sigma-Aldrich. Phosphate buffered saline (PBS) was purchased from Oxoid Limited (Hampshire, UK). 12-O-Tetradecanoylphorbol 13-acetate (TPA), dimethyl sulfoxide (DMSO), lipopolysaccharides (LPS) from *Escherichia coli* O111:B4 purified by phenol extraction and 3-(4,5-dimethyl-2-thiazolyl)-2,5-diphenyl-2*H*-tetrazolium bromide (MTT) were purchased, from Sigma-Aldrich.

Dihydrochalcone standards, phlorizin and phloretin, were purchased from Sigma-Aldrich and were dissolved in DMSO, each at a stock concentration of 100 mM. The final concentration of DMSO in culture was ≤ 0.1%.

Standards of D,L-norvaline, succinic acid-2,2,3,3-d_4_ and *trans*-cinnamic-d_7_ acid, used as surrogates, were purchased from Sigma-Aldrich. Each standard was prepared at 100 mM in water or methanol depending on the solubility of the chemical. Surrogate solution and a mix of standards were prepared in methanol at 1.0 mM. These solutions were stored in amber flasks at −20 °C.

In order to correct injection disturbances during GCMS determinations, two polychlorinated biphenyls (PCB30 and PCB204), purchased from Accustandard (New Haven, CT, USA), were used as internal standard (IS). Saturated Alkane Mixture (C_7_–C_40_) and FAME mix solution were acquired from Supelco (Bellefonte, PA, USA) to support the metabolite identification. Working solutions of *n*-alkanes and FAMEs mixture was prepared in acetone at 1.0 mg L^−1^. For derivatization: *N*-methyl-*N*-trimethylsilyl trifluoroacetamide (MSTFA), chlorotrimethylsilane (TMCS) and pyridine were purchased from Sigma Aldrich. Methoxylamine hydrochloride (MeOX) was from Supelco.

### 3.2. In Vitro Assay

#### 3.2.1. Culture of THP-1 Cells and Their Maturation into Macrophages

The human monocytic THP-1 cell line was cultured in suspension at a density of 3.0 × 10^5^ cells mL^−1^ in RPMI 1640 medium supplemented with 10% FBS and 1.0% of an antibiotic/antimycotic solution; and maintained in an incubator with humidified atmosphere, 5.0% CO_2_ at 37 °C.

A total of 2.0 × 10^6^ cells mL^−1^ were seeded into 12-well plates using 2.0 mL cell suspension per well. Then, monocytic THP-1 cells were differentiated into mature macrophage-like cells by stimulating with 25 nM 12-O-tetradecanoylphorbol 13-acetate (TPA) for 48 h. Non-attached cells were removed by aspiration and adherent cells were washed three times with PBS, followed by 24 h rest in the absence of TPA with fresh medium as was proposed by Lund et al. [27] to induce a consistent phenotype of THP-1 macrophage for the study of inflammatory responses. Cells were checked under a light microscope for the evidence of differentiation.

#### 3.2.2. Co-Cultivation of Dihydrochalcones and THP1 Cells

After 24 h rest, cells were incubated with final concentration of 100 μM of each dihydrochalcone (PZ and PT) as the concentration giving the most significant response in previous studies [7], with and without LPS (1.0 μg mL^−1^). Also untreated and LPS-stimulated THP-1 macrophages, without the presence of test compounds, were assayed. As well as, media for both control groups contained 0.10% DMSO, to match the experimental groups. In addition, blank samples (with no cells) were included in the study. All conditions were tested in triplicate. The microplates were incubated for 2 days at 37 °C with 5.0% CO_2_.

#### 3.2.3. Sample Preparation for Untargeted Metabolomics

After 48 h culture, macrophage-like THP-1 cells were submitted to a quenching protocol to avoid degradation of labile metabolites. Briefly, according to previous metabolomics study [13], plates were transferred to ice and culture medium was completed removed. Then, 20 μL of surrogate standards solution followed by 800 μL of chloroform:methanol:water (1:3:1, *v*/*v*) were added to cells. After incubation for 1.0 h, at 4.0 °C in an oscillating stirrer, cell suspensions were transferred to cold microcentrifuge tubes for supernatants harvesting (13,000× *g*, 3.0 min, 4.0 °C), where intracellular metabolites were present.

For LC-MS analysis, 400 μL of supernatants were reduced to dryness under gentle nitrogen stream and redissolved in 200 μL acetonitrile:water (1:9, *v*/*v*).

For GC-MS analysis, derivatization process is required. According to Cambeiro-Pérez et al. [12], the remaining 400 μL of quenching extracts were reduced to dryness under a gentle nitrogen stream and then derivatized in two steps. Firstly, the dried samples were dissolved in 100 μL of MeOX (20 mg mL^−1^ in pyridine) for methoxymation, vortexed for 1.0 min and incubated at room temperature for 1 h. Secondly, 100 μL MSTFA with 1.0% TMCS was added to samples for silylation followed by incubation at 70 °C for 30 min. Finally, derivatized extracts were transferred to vials and spiked with 2.0 μL of IS mix solution (100 mg L^−1^) for GC-MS analysis.

#### 3.2.4. Quality Control Samples

For controlling the quality of metabolomics data, three different types of Quality Control (QC) samples have been employed: (i) Biological QC sample (QC) was made by pooling 45 μL from each of analysed samples, (ii) Surrogate standard QC sample (QC surrogate) was made by spiking with surrogate standards to the QC sample and (iii) Reference standard QC sample was prepared with a mix of standards to evaluate the derivatization process, as was reported previously by our research group [12,13].

### 3.3. Multiplatform Analysis

#### 3.3.1. LC-HRMS

The LC system consisted of an Agilent (Wilmington, DE, USA) 1200 Series LC system interfaced to an Agilent 6520 Q-TOF mass spectrometer equipped with a dual electrospray ionization (ESI) source operating in both positive and negative ion modes. The instrument was interfaced to a PC computer running the Agilent MassHunter Workstation Software B.07.00. The Q-TOF instrument was operated in extended dynamic range 2.0 GHz mode. The parameters of the mass spectrometer under the ESI mode were: nebulizer (N_2_), 42 psig; drying gas (N_2_) flow rate, 5.0 L min^−1^; drying gas temperature, 350 °C; and fragmentor voltage, 120 V. To obtain high-accuracy mass measurements, reference calibration solution (Agilent Technologies, Santa Clara, CA, USA) was continuously applied. Chromatographic separation was performed on a Kinetex^TM^ 2.6 μm Polar C18 column (100 Å, LC Column 100 × 2.1 mm) (Phenomenex, Torrance, CA, USA) and the temperature of column was maintained at 35 °C. The mobile phase consisted of 0.10% formic acid in water (A) and 0.10% formic acid in acetonitrile (B). The gradient program was as follows: 100% A during 2.0 min, change to 100% B in 18 min and hold during 5.0 min, change to initial conditions in 0.10 min and hold for 10 min, giving an analysis time of 35 min. The flow rate was set at 0.20 mL min^−1^ and the injection volume was 5.0 μL.

#### 3.3.2. GC-MS

The GC system consisted of an Agilent 7890 gas chromatograph coupled to an Agilent 7000C triple quadrupole detector and to an Agilent 7650C autosampler. MassHunter Acquisition Software B.08.00 version (Agilent Technologies) was used to control the equipment and for data acquisition. Each derivatized sample (0.5 μL) was injected in splitless mode at 280 °C using a multimode inlet (MMI) equipped with an Ultra Inert liner from Agilent. Chromatographic separation was done by using an Agilent HP 5MS capillary column (30 m × 0.25 mm × 0.25 μm). The oven temperature was initially 100 °C (4.0 min hold) and ramped to 300 °C at 4.0 °C min^−1^ (1.56 min hold). Helium was used as a carrier gas with a flow rate of 1.0 mL min^−1^. The MS was operated in electron impact ionization mode at 70 Ev and full scan monitoring mode from *m*/*z* 35 to 600. The transfer line and ionization source temperatures were set to 280 °C.

### 3.4. Multiplatform Data Processing, Metabolite Identification and Statistical Analysis

The resulting raw data files (.d files) were evaluated through visual inspection of Total ion chromatograms (TICs) to check the quality of the analytical run using the MassHunter Qualitative Analysis B.07.00 (Agilent Technologies). Then, data processing was firstly performed independently based on the analytical platform used for sample acquisition.

On the one hand, LC-MS samples were pre-processed as was reported by Cambeiro-Pérez et al. [13] using MassHunter Profinder Software B.08.00 (Agilent Technologies), with Batch Recursive Feature Extraction (BRFE) algorithm using the following parameters: minimum peak height of 150 counts; tolerance window for compound binning and alignment of 0.50 min for retention time and a mass accuracy of 20 ppm for *m/z* values; and set a minimum absolute height of 1000 counts for peak filtering. Next, extracted compounds were exported as compound exchange files (.CEF) for feature alignment, data processing, compound identification and preliminary statistical analysis using Mass Profiler Professional (MPP) B.14.08 (Agilent Technologies). Data were filtered with a retention time tolerance of 0.15 min; mass tolerance of 2.0 mDa; *p*-value < 0.05; and fold change > 2. Statistically significant metabolites were putatively identified through MassHunter METLIN Metabolite PCDL (Agilent Technologies) and unknown metabolites were identified by their molecular formulas.

On the other hand, GC-MS samples were pre-processed as was reported by Cambeiro-Pérez et al. [12] using MassHunter Unknowns Analysis Tool B.07.01 (Agilent Technologies) for deconvolution and identification. Metabolites putative identification was performed according to a QC in-house spectral library, created previously by comparison of the mass spectra with NIST 11 (National Institute of Standards and Technology, 2011) and Fiehn RTL (Agilent Technology) libraries at 70% similarity index. To support the identification, both n-alkanes and FAMEs mixtures were analysed during the analytical batch for retention indices determination. Then, data were aligned and normalized prior to statistical analysis, using MPP and filtered: by frequency, retaining those entities which appeared in more than 100% of samples in at least one condition; *p*-value < 0.05; and fold change > 2.

Finally, entities list was exported for both LC and GC from MPP after filtering to create a combined entity list for further multivariate statistical analysis. One-way ANOVA with Tukey’s honest significance difference (HSD) post Hoc test was performed by MPP to identify which entities were responsible for significant differences among groups with a *p*-value < 0.050. Fold change cut-off > 2.0 was also applied. Moreover, unsupervised principal component analysis (PCA) and hierarchical cluster analysis (HCA) were performed for data exploration and visualization, providing information about the presence of outliers, sample dispersion and data clustering. Samples were classified into discrete classes also by supervised partial least square discriminant analysis (PLS-DA).

### 3.5. Pathways Interpretation

Biological interpretation was performed by MetaboAnalyst 4.0 [16] through pathway analysis tool and the metabolite set library *Homo sapiens* based on normal human metabolic pathways from the Kyoto Encyclopedia of Genes and Genomes (KEGG) database [28]. Moreover, other freely available online databases were employed as the Human Metabolome Database (HMDB) [29] and LIPID MAPS^®^ [30].

## 4. Conclusions

This study provides an untargeted multiplatform metabolite profiling approach to understand metabolic effects promoted by phlorizin and phloretin, under normal and inflammatory conditions, in human macrophage-like THP-1 cells by LC-QTOF-MS and GC-MS. Results suggest differential phenotypic response in macrophages treated with LPS, PZ and PT. Moreover, since dihydrochalcone stimulation under inflammatory environment mimics the response of these flavonols under normal conditions, it might suggest that dihydrochalcones were capable of inhibiting the response triggered by LPS. Furthermore, antagonistic effects of dihydrochalcones against the pro-inflammatory metabolism promoted by LPS was mainly observed in glycerophospholipid and sphingolipid metabolism in addition to promoting amino acid biosynthesis. Nevertheless, PT seems to exert greater immunomodulatory capacity than PZ, which showed limited metabolic alterations in comparison to untreated THP-1 derived macrophages. Due to macrophages heterogeneity, it could be relevant to study in vivo metabolism for a better understanding dihydrochalcone glycosylated/aglycone forms bioavailability and real immunomodulatory mechanisms.

## Data Availability

The data presented in this study are available on request from the corresponding authors.

## Supplementary Materials

The following are available online, Table S1: Significantly changed metabolites (FC > 2.0 and *p*-value ≤ 0.05) within THP-1 cells treated with lipopolysaccharide (LPS); pure dihydrochalcones (phlorizin (PZ), phloretin (PT)); and pure dihydrochalcones with LPS (PZ + LPS (PZL) and PT + LPS (PTL)); in comparison with untreated controls (Basal). Results were expressed in terms of log FC [(Treatment) vs (Basal)], representing the most significant metabolic pathways and its putative metabolites.

## Sample Availability

Samples of the compounds are unavailable from the authors.
