# Supplementary material for: Metabolomics Insights of the Immunomodulatory Activities of Phlorizin and Phloretin on Human THP-1 Macrophages
Source: Molecules. 2021 Feb 3;26(4):787. doi: 10.3390/molecules26040787 (PMC7913540; doi:10.3390/molecules26040787)
Supplement: Supplementary file 1 [file molecules-26-00787-s001.pdf]

Table S1. Significantly changed metabolites (FC > 2.0 and p-value ≤ 0.05) within THP-1 cells treated with lipopolysaccharide (LPS); pure dihydrochalcones (phlorizin (PZ), phloretin (PT)); and pure dihydrochalcones with LPS (PZ+ LPS (PZL) and PT+LPS (PTL)); in comparison with untreated controls (Basal). Results were expressed in terms of Log FC [(Treatment) vs (Basal)], representing the most significant metabolic pathways and its putative metabolites.

Compound	System	RT	Mass	LPS	PZ	PT	PZL	PTL
Aminoacyl-tRNA biosynthesis								
D-aspartate	LC	30.22	176.9992			-20.752		-20.416
Glycine	GC	6.73	75	10.174	10.771	9.384	11.458	12.698
L-asparagine	GC	15.13	73			9.679	9.783	12.817
L-isoleucine	GC	9.61	86	7.735	6.768	6.332	8.847	10.146
L-leucine	GC	15.01	158	7.292		11.215	14.274	16.684
L-glutamate	GC	15.66	246	6.491	3.162	13.365	15.719	16.041
L-phenylalanine	GC	16.20	73	9.423	3.162	9.056	12.524	12.435
L-proline	GC	9.84	70	12.688				
L-threonine	GC	11.51	73	6.559	5.458	7.148	8.652	7.458
L-valine	GC	15.29	73	18.236		16.679	16.875	13.774
Valine, leucine and isoleucine biosynthesis								
Glycine	GC	6.73	75	10.174	10.771	9.384	11.458	12.698
L-isoleucine	GC	9.61	86	7.735	6.768	6.332	8.847	10.146
L-leucine	GC	15.01	158	7.292		11.215	14.274	16.684
L-leucine	GC	15.01	158	7.292		11.215	14.274	16.684
L-threonine	GC	11.51	73	6.559	5.458	7.148	8.652	7.458
L-valine	GC	15.29	73	18.236		16.679	16.875	13.774
Alanine, aspartate and glutamate metabolism								
D-aspartate	LC	30.22	176.9992			-20.752		-20.416
L-asparagine	GC	15.13	73			9.679	9.783	12.817
L-glutamate	GC	15.66	246	6.491	3.162	13.365	15.719	16.041
N-acetyl-L-aspartate	GC	17.46	73		5.537	13.163	13.019	13.587
N-methylglutamate	GC	15.22	260		3.359	15.631	12.647	15.736

Table S1. Continued.

**Compound**	**System**	**RT**	**Mass**	**LPS**	**PZ**	**PT**	**PZL**	**PTL**
Arginine biosynthesis								
Urea	GC	11.09	147	7.726	3.242	14.155	17.268	17.611
L-glutamate	GC	15.66	246	6.491	3.162	13.365	15.7186	16.041
Glycerophospholipid metabolism								
Dihydroxyacetone phosphate	GC	17.15	73	12.210				
Glycerol 1-phosphate	GC	17.34	73	7.176				
LysoPE(18:1(11Z)/0:0) 19.980001	LC	19.98	479.3006	-1.472				
LysoPE(20:4(8Z,11Z,14Z,17Z)/0:0)	LC	19.10	501.2839	-1.124				
O-phosphoethanolamine	GC	17.62	73	12.784	-2.838		1.443	1.702
PE(14:0/21:0)[U]	LC	29.84	733.5603		-3.708	-18.053	-1.699	-18.433
PE(16:0/20:1(11Z))	LC	29.84	762.5902				-16.711	-16.824
PE(18:1(9Z)/0:0)	LC	19.29	501.2800		13.559		13.559	
PE(18:1(9Z)/20:0)[U]	LC	29.84	773.6003		-14.272	-14.438	-14.553	-14.879
PE(19:0/0:0)	LC	23.80	495.3360			15.318		
PE(21:0/0:0)	LC	21.40	523.362	1.891				
PE(22:4(7Z,10Z,13Z,16Z)/17:0)	LC	29.69	781.568	1.080				
PG(14:0/15:0)	LC	25.84	697.4879			1.633	2.817	1.452
PG(14:0/15:0) Esi+26.904219	LC	26.90	697.4886			3.509		3.303
PG(15:0/12:0)	LC	25.86	669.4590			1.181		
PG(15:0/12:0) Esi+25.798006	LC	25.80	669.4593			14.688		14.286
PG(15:0/18:2(9Z,12Z))	LC	23.77	732.4918	1.239				
PG(15:0/16:0)	LC	26.94	725.5173	-5.161		1.873		1.795
PG(18:0/0:0)	LC	21.10	529.3346					13.66
PC(18:0/18:1(6Z))	LC	29.84	787.6075		-1.723			
PC(18:1(17Z)/18:1(17Z))	LC	29.83	785.5922		-3.154	-17.522	-8.267	-8.462
PC(19:1(9Z)/15:0)	LC	29.83	759.5754		-1.654	-18.993	-18.259	-19.287
PG(20:5(5Z,8Z,11Z,14Z,17Z)/0:0)	LC	22.20	530.2640			1.887		1.982

Table S1. Continued.

Compound	System	RT	Mass	LPS	PZ	PT	PZL	PTL
Glycerophospholipid metabolism								
PG(20:3(8Z,11Z,14Z)/15:0)	LC	23.75	758.507	-1.306				
PG(22:4(7Z,10Z,13Z,16Z)/15:1(9Z))	LC	23.75	782.5088	-1.396				
PS(22:1(11Z)/17:0)	LC	24.16	831.6009	1.720				
PS(22:4(7Z,10Z,13Z,16Z)/22:1(11Z))	LC	19.84	893.6108	-10.279				
PS(P-20:0/16:0)	LC	20.83	775.5722	-3.236				
sn-glycero-3-phosphocholine	LC	1.42	257.1026	1.619				-15.932
Sphingolipid metabolism								
Cer(d16:1/18:0)	LC	23.82	559.4912	2.743				1.403
Cer(d16:1/18:0) 25.460001	LC	25.46	537.5093	-2.568				
Cer(d16:2(4E,6E)/20:1(11Z))	LC	23.57	583.4890					14.855
Cer(d16:1/20:0)	LC	27.09	565.5392	-1.576				
Cer(d16:2(4E,6E)/18:0)	LC	24.69	535.4931	3.245				
Cer(d18:0/12:0)	LC	23.70	483.4590	-2.580		-17.287		
Cer(d18:0/12:0) Esi+23.715666	LC	23.72	483.4620			17.812		17.829
Cer(d18:0/14:0)	LC	24.87	511.4968	-3.844				
Cer(d18:0/14:0) 25.700006	LC	25.70	511.4966	8.228				
Cer(d18:0/14:0) 26.140007	LC	26.14	511.4958	3.400				
Cer(d18:0/16:0)	LC	27.07	539.5278	-2.905				
Cer(d18:0/16:0) 26.459995	LC	26.46	539.5278	2.473				
Cer(d18:0/16:0) 27.549995	LC	27.55	539.5266	-2.925				
Cer(d20:0/16:0)	LC	28.32	567.5546				-2.445	1.073
NeuGcalpha2-3Galbeta1-4GlcNAcbeta1-3Galbeta1-4GlcNAcbeta1-3Galbeta1-4Glcbeta-Cer(d18:1/16:0)	LC	22.51	1942.9373			1.949		4.372
O-phosphoethanolamine	GC	17.62	73	12.784	-2.838	1.667	1.443	1.702
Phytosphingosine	LC	17.84	317.2906					16.862
